# Design of a Metasurface with Long Depth of Focus Using Superoscillation

**DOI:** 10.3390/nano13182500

**Published:** 2023-09-05

**Authors:** Tianyu Zhao, Xiao Lv, Yue Wang, Yihui Wu

**Affiliations:** 1 Changchun Institute of Optics, Fine Mechanics and Physics (CIOMP), Chinese Academy of Sciences, Changchun 130033, China; zhaotianyu@ciomp.ac.cn (T.Z.); lvxiao@ciomp.ac.cn (X.L.); wangyue@ciomp.ac.cn (Y.W.); 2 GD Changguang Zhongke Bio Co., Ltd., Foshan 528200, China

**Keywords:** metalens, extended focal depth, polarization-independent

## Abstract

Longitudinal optical field modulation is very important for applications such as optical imaging, spectroscopy, and optical manipulation. It can achieve high-resolution imaging or manipulation of the target object, but it is also limited by its depth of focus. The depth of focus determines whether the target object can be clearly imaged or manipulated at different distances, so extending the depth of focus can improve the adaptability and flexibility of the system. However, how to extend the depth of focus is still a significant challenge. In this paper, we use a super-oscillation phase modulation optimization method to design a polarization-independent metalens with extended focal depth, taking the axial focal depth length as the optimization objective. The optimized metalens has a focal depth of 13.07 μm (about 22.3 λ), and in the whole focal depth range, the transverse full width at half maximum values are close to the Rayleigh diffraction limit, and the focusing efficiency is above 10%. The results of this paper provide a new idea for the design of a metalens with a long focal depth and may have application value in imaging, lithography, and detection.

## 1. Introduction

To improve the performance of imaging systems, precise manipulation of the optical field is required. The optical field produces longitudinal field components when it varies along the propagation direction; these components have extraordinary optical manipulation capabilities. In the past few decades, people have extensively studied the longitudinal field components. For example, when the focusing of radial polarization light is used to enhance longitudinal field components, a special beam, namely a longitudinal polarization light needle [1,2], can be formed. This light needle has potential application prospects in particle acceleration and fluorescent particle capture [3,4,5].

However, one of the challenges in optical imaging is to achieve a high resolution and a long depth of field simultaneously. The depth of field is the distance range over which the image is in focus. A long depth of field is desirable for capturing clear images of objects at different distances from the lens. However, conventional lenses have a trade-off between resolution and depth of field: increasing the numerical aperture (NA) of the lens can improve the resolution, but it also reduces the depth of field. Therefore, there is a need for novel lenses that can overcome this limitation and achieve both high resolution and long depth of field.

The methods for achieving extended focal depth that have been reported so far include Fresnel lens arrays (FLAs), axial lenses (AXLs) and light sword optical elements (LSOEs) [6,7]. However, due to the difficulty of manufacturing traditional lenses, they are at a significant disadvantage in the development of extended focal depth lenses.

Subwavelength metasurfaces [8,9,10,11,12,13,14,15,16,17,18,19,20,21,22,23,24,25,26,27,28,29,30,31,32,33,34,35,36], which can arbitrarily control the amplitude, phase, and polarization characteristics of the incident light, thus achieving new functions that traditional lenses cannot achieve, have become a research hotspot in the current optical field. In the process of realizing the metasurface function, commonly used finite focal depth metasurfaces and extended focal depth light sword metasurfaces have some limitations, such as polarization sensitivity caused by geometric phase structures or design parameters that are not flexible enough due to forward design thinking. To improve these limitations, a more freely designed, polarization-independent metasurface design method needs to be explored.

In this paper, we design a polarization-insensitive metasurface based on titanium dioxide dielectric cylindrical unit structures, and use an innovative super-oscillation phase modulation method, which enables the metasurface to achieve near-diffraction-limit focusing performance and 22.3 λ extended focal depth under any polarization light excitation. The metasurface design method overcomes the limitations of traditional geometric phase structures, providing subwavelength metasurfaces with greater design freedom and stronger functionality. The metasurface has long focal depth and polarization-insensitive characteristics and has broad application prospects in imaging, lithography, and detection.

## 2. Materials and Methods

**Figure 1:**
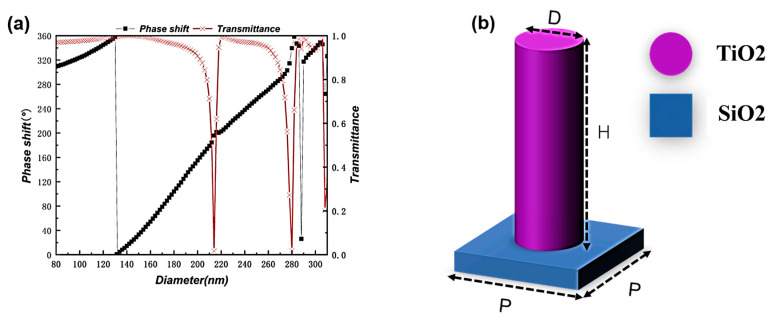
(**a**) shows the phase distribution of the unit structure (**b**) at a wavelength of 587 nm when the period, P, is 320 nm; (**b**) shows the schematic diagram of the unit structure, where the substrate material is SiO_2_ and the unit structure is TiO_2_, with a height of 600 nm and a period of 320 nm.

The phase shift of the metasurface designed in this paper, which consists of cylindrical dielectric nanocolumn structures with rotational symmetry, depends on the transmission phase with polarization-independent characteristics. Therefore, in order to match the ideal phase obtained by calculation, we need to construct a phase library of nanocolumns. We use the commercial software package “FDTD Solutions” (ANSYS Inc. (Lumerical Inc., Vancouver, BC, Canada) to perform finite-difference time-domain (FDTD) simulation of nanocolumns and obtain the phase shift of nanocolumns when each geometric parameter increases by 2 nm. Periodic boundary conditions are applied along the x and y directions, and perfectly matched layers (PMLs) are applied along the z direction. The height, H, is fixed at 600 nm; the scanning range of the diameter of the nanocolumns is 80–310 nm; and the period of the properly arranged nanocolumn array, P, is 320 nm. As can be seen from Figure 1a, when P = 320 nm, the obtained propagation phase library can cover the phase distribution, meeting our requirements for phase matching.

In this paper, we first design the phase function of the super-oscillation lens. The phase function of the device can be divided into two parts: the focusing phase function $\Phi_{\mathrm{lens}\;}\left(r\right)$ and the superoscillation phase modulation function $\Phi_{SO}\left(r\right)$.

**Figure 2:**
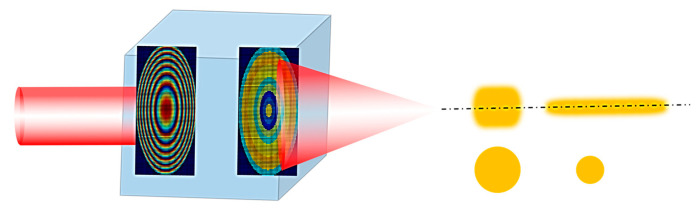
Metasurface design schematic diagram.

The schematic diagram of the design in this paper is shown in Figure 2. By introducing and adjusting the super-oscillation phase, the energy at the original focal point is stretched along the axis. While achieving the purpose of long focal depth, this also reduces the lateral spot size and improves the resolution.

The first part is the focusing phase, which can focus the optical field. This part of the phase function can be expressed as:

(1) $$\Phi_{\mathrm{lens}\;}\left(r\right)=\frac{2\pi }{\lambda_{0}}\left(-\sqrt{f_{0}^{2}+r^{2}}+f_{0}\right)+2m\pi$$

where $\lambda_{0}$ is the design center wavelength; $f_{0}$ is the focal length; m is an integer; r is the radius of the lens structure. The designed superoscillation lens structure has a diameter of 32 μm and a focal length of 30 μm.

The super-oscillation modulation phase function $\Phi_{SO}\left(r\right)$ can achieve long focal depth focusing by modulating the size of the high-frequency and low-frequency spatial spectrum based on the focused light field.

The specific design principle: using the point source method to calculate the intensity distribution of the axial diffraction light field, taking the depth of the focus (axial half-height full-width) length as the objective function and then selecting the appropriate structure from the unit structure database obtained from simulation via the optimization algorithm. The formula for calculating the light field distribution via the point source method can be expressed using the following formula [37]:

(2) $$E\left(x_{0},y_{0},z_{0}\right)=\overset{N-1}{\underset{j=0}{\sum }}\frac{A_{j}}{r_{j}}\exp\left[i\left(kr_{j}+\phi_{j}\right)\right]$$

where$\;r_{j\;}$represents the distance between the metasurface unit and the focal point $\left(x_{0},y_{0},z_{0}\right)$, N is the total number of metasurface units, $A_{j}$ is the amplitude intensity of the unit structure (here replaced by the structure’s transmittance), $\phi_{j}$ is the phase response of the metasurface unit structure. The formula is composed of structures selected from the transmission phase database that match φ, and $E\left(x_{0},y_{0},z_{0}\right)$ is the electric field intensity at point $\left(x_{0},y_{0},z_{0}\right)$.

(3) $$\varphi =\Phi_{\mathrm{lens}\;}\left(r\right)+\Phi_{SO}\left(r\right)$$

**Figure 3:**
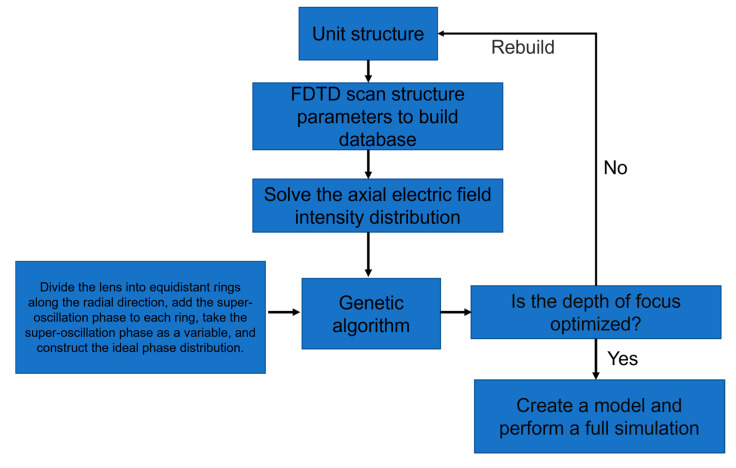
Long focal depth metasurface optimization flow chart.

The specific optimization solution process is shown in Figure 3:

As shown in the flow chart: First, a database of the transmission phase and transmittance of the unit structure needs to be established, and then the designed metasurface is divided into N equidistant rings along the radial direction; N is 10 in this paper. Then, the phase variables in the optimization algorithm are added to each ring to construct a new ideal phase distribution. The optimization algorithm can choose any one of these: particle swarm optimization algorithm, cuckoo search algorithm, simulated annealing algorithm, genetic algorithm, etc. This paper uses the genetic algorithm.

## 3. Results

**Figure 4:**
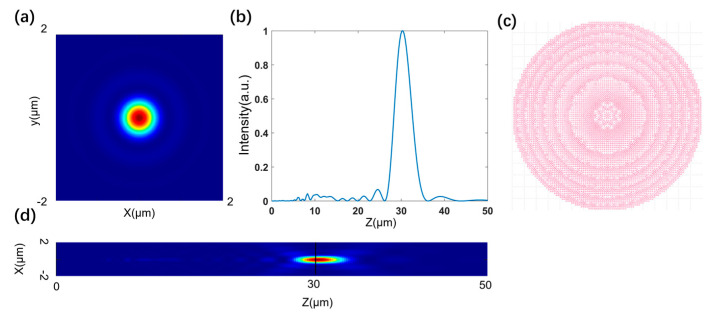
(**a**) Transverse electric field intensity distribution of metasurface when $f_{0}=30\;\mu m$. (**b**) Distribution curve of axial electric field intensity of metasurface. (**c**) gds layout of metasurface stru-ture. (**d**) Axial electric field distribution diagram of metasurface.

**Figure 5:**
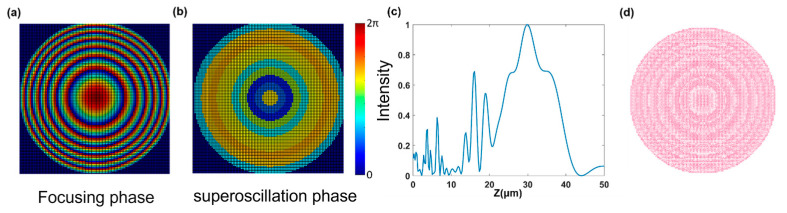
(**a**) The original focusing phase of the metasurface. (**b**)The optimized super-oscillation phase of the metasurface. (**c**) Axial field distribution curve of metasurface. (**d**) gds layout of metasurface structure.

**Figure 6:**
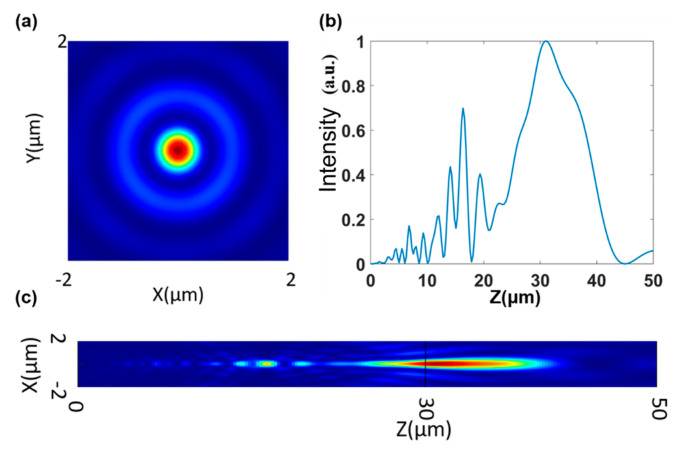
(**a**) Transverse electric field intensity distribution of metasurface when $f_{0}=30\;\mu m$. (**b**) Distribution curve of axial electric field intensity of metasurface. (**c**) Axial electric field distribution diagram of metasurface.

Using phase matching, we select the appropriate unit structure from the database and place it at the corresponding position of the metasurface to construct the complete structure of the metasurface. We use the point source method to solve the axial electric field intensity distribution of the metasurface and take the axial electric field depth of focus as the optimization objective function. At the same time, in order to ensure the position of the focal point, we set the position of the maximum value as 30 μm as a constraint condition to ensure the rationality of the design. Then, using continuous iterative optimization, we obtain the corresponding parameters of the design. For comparison, we first use a metasurface structure without super-oscillation phase modulation for calculation and obtain the simulation results as shown in Figure 4:

When the superoscillation phase modulation is added, the optimized result is shown in Figure 5:

After obtaining the corresponding design parameters, the full-wave simulation software FDTD Solutions was used to verify and obtain the corresponding results as shown in the following figure:

By comparing the axial electric field intensity distribution curves of Figure 5c and Figure 6b, it can be seen that the general trends of the two are almost consistent when the DOF (depth of focus) value of Figure 5c is 14.57 μm and the DOF value of Figure 6b is 13.07 μm, which are also close. The main reason for the error is that the point source method cannot account for the coupling between structures when calculating the light field, as the full-wave simulation does, but compared with other diffraction calculation theories, this algorithm has a certain advantage in accuracy because it particularly considers the influence of the amplitude transmittance of the structure. Figure 6a shows the lateral electric field intensity distribution of the structure at $f_{0}=30\mu m$, and after calculation, its full width at half maximum value is 616 nm, which is less than the Rayleigh diffraction limit, and the focusing efficiency is 28.4%. By observing the axial electric field intensity distribution in Figure 6c, it can be seen that the focused light field in the whole depth of focus range becomes very small in lateral size due to longitudinal stretching, the lateral full width at half maximum values are all less than the diffraction limit, and the focusing efficiency is also above 10%. The specific simulation results parameters are shown in Table 1:

When the modulation is not performed with the super-oscillation phase, the depth of focus of the metasurface is 4.3 μm, the depth of focus is about three times greater after adding the super-oscillation phase modulation, and the resolution is also improved.

## 4. Conclusions

This paper proposes a polarization-independent long focal depth metasurface structure using the super-oscillation phase optimization method. By combining the focusing phase and the super-oscillation phase as optimization variables, the focal depth of the metasurface is successfully increased to 13.07 μm (about 22.3 λ). The lateral full width at half maximum values in the whole focal depth range are close to the Rayleigh diffraction limit, and the focusing efficiency is above 10%. At the same time, this method uses the data in the fixed unit structure; unlike the topology optimization method, it does not need to change the shape of the structure, has more advantages in processing, and meets the conditions for processing and manufacturing. Compared with the traditional forward design style of long focal depth metasurface, the design method in this paper has a greater degree of freedom and is easier to control in terms of the relevant performance of the metasurface.

The results of this paper provide a new perspective for the design of long focal depth metasurface and may find potential applications in imaging, holography and optical manufacturing.

## Figures and Tables

**Table 1:** Axial focal position, focusing efficiency, and lateral full width at half maximum(FWHM).

Axial Focal Position (μm)	Focusing Efficiency (%)	FWHM (μm)
25.73	11.81	472
30	28.4	616
30.95	29.44	664
38.79	21.63	888

## Data Availability

Not applicable.

## References

[1] Wang H., Shi L., Lukyanchuk B., Sheppard C., Chong C.T. (2008). Creation of a Needle of Longitudinally Polarized Light in Vacuum Using Binary Optics. Nat. Photonics.

[2] Yu A., Chen G., Zhang Z., Wang H., Shi L. (2016). Creation of Sub-Diffraction Longitudinally Polarized Spot by Focusing Radially Polarized Light with Binary Phase Lens. Sci. Rep..

[3] Gupta D.N., Kant N., Kim D.E., Singh K.P. (2007). Electron Acceleration to GeV Energy by a Radially Polarized Laser. Phys. Lett. A.

[4] Cicchitelli L., Hora H., Postle R. (1990). Longitudinal Field Components for Laser Beams in Vacuum. Phys. Rev. A.

[5] Zhan Q. (2004). Trapping Metallic Rayleigh Particles with Radial Polarization. Opt. Express.

[6] Petelczyc K., Bará S., Lopez A.C., Jaroszewicz Z., Kolodziejczyk A., Sypek M. (2011). Imaging Properties of the Light Sword Optical Element Used as a Contact Lens in a Presbyopic Eye Model. Opt. Express.

[7] Kakarenko K., Ducin I., Grabowiecki K., Jaroszewicz Z., Kolodziejczyk A., Sypek M. (2015). Assessment of Imaging with Extended Depth-of-Field by Means of the Light Sword Lens in Terms of Visual Acuity Scale. Biomed. Opt. Express.

[8] Yu N., Genevet P., Kats M.A., Aieta F., Tetienne J.P., Capasso F., Gaburro Z. (2011). Light Propagation with Phase Discontinuities: Generalized Laws of Reflection and Refraction. Science.

[9] Huang L., Chen X., Muhlenbernd H., Li G., Bai B., Tan Q., Jin G., Zentgraf T., Zhang S. (2012). Dispersionless Phase Discontinuities for Controlling Light Propagation. Nano Lett..

[10] Sun S., He Q., Xiao S., Xu Q., Li X., Zhou L. (2012). Gradient-Index Meta-Surfaces as a Bridge Linking Propagating Waves and Surface Waves. Nat Mater.

[11] Yin X., Ye Z., Rho J., Wang Y., Zhang X. (2013). Photonic Spin Hall Effect at Metasurfaces. Science.

[12] Luo X.G. (2015). Principles of Electromagnetic Waves in Metasurfaces. Sci. China Phys. Mech. Astron..

[13] Grady N.K., Heyes J.E., Chowdhury D.R., Zeng Y., Reiten M.T., Azad A.K., Chen H.T., Taylor A.J., Dalvit D.A.R., Chen W.T. (2013). Terahertz Metamaterials for Linear Polarization Conversion and Anomalous Refraction. Science.

[14] Zheng G., Mühlenbernd H., Kenney M., Li G., Zentgraf T., Zhang S. (2015). Metasurface Holograms Reaching 80% Efficiency. Nat. Nanotechnol..

[15] Wen D., Yue F., Li G., Zheng G., Chan K.F.L., Chen S., Chen M., Li K.F., Wong P.W.H., Cheah K.W. (2015). Helicity Multiplexed Broadband Metasurface Holograms. Nat. Commun..

[16] Chen W.T., Yang K.Y., Wang C.M., Huang Y.W., Sun G., Chong C.T., Tsai D.P. (2014). High-Efficiency Broadband Meta-Hologram with Polarization-Controlled Dual Images. Nano Lett..

[17] Ye W., Zeuner F., Li X., Reineke B., He S., Liu W., Wang Z., Wang F., Zhang S., Chen S. (2016). Spin and Wavelength Multiplexed Nonlinear Metasurface Holography. Nat. Commun..

[18] Zhang F., Pu M., Li X., Ma X., Luo X., Guo J., Hu C., Wang Z., Zhao Z., Wang C. (2017). All-Dielectric Metasurfaces for Simultaneous Giant Circular Asymmetric Transmission and Wavefront Shaping Based on Asymmetric Photonic Spin–Orbit Interactions. Adv. Funct. Mater..

[19] Arbabi A., Horie Y., Bagheri M., Faraon A. (2015). Dielectric Metasurfaces for Complete Control of Phase and Polarization with Subwavelength Spatial Resolution and High Transmission. Nat. Nanotechnol..

[20] Pu M., Li X., Ma X., Wang Y., Zhao Z., Wang C., Luo X. (2015). Catenary Optics for Achromatic Generation of Perfect Optical Angular Momentum. Sci. Adv..

[21] Li X., Chen L., Li Y., Zhang Z., Sabat R.G., Lin J.T.J., Genevet P., Capasso F., Zhang S., Chen X. (2016). Multicolor 3D Meta-Holography by Broadband Plasmonic Modulation. Sci. Adv..

[22] Wang B., Dong F., Li Q.T., Yang D., Sun C., Chen W.T., Zhang S., Chen X. (2016). Visible-Frequency Dielectric Metasurfaces for Multiwavelength Achromatic and Highly Dispersive Holograms. Nano Lett..

[23] Chen B.H., Wu P.C., Su V.C., Liao W.Y., Chen T.Y., Huang T.T., Wang J.W., Sun G., Tsai D.P. (2017). GaN Metalens for Pixel-Level Full-Color Routing at Visible Light. Nano Lett..

[24] Jin L., Dong Z., Mei S., Yu Y.F., Wei Z., Pan W., Kivshar Y.S., Zhang S. (2018). Noninterleaved Metasurface for (26-1) Spin-and Wavelength-Encoded Holograms. Nano Lett..

[25] Zang X.F., Gong H.H., Li Z., Wang Y., Zhang C., Yue F., Wang C., Luo X. (2018). Metasurface for Multi-Channel Terahertz Beam Splitters and Polarization Rotators. Appl. Phys. Lett..

[26] Mehmood M.Q., Mei S., Hussain S., Lee K.H., Zhang W., Tripathy S., Qiao S., Danner A.J., Zhang S. (2016). Visible-Frequency Metasurface for Structuring and Spatially Multiplexing Optical Vortices. Adv. Mater..

[27] Yue F., Zhang C., Zang X.F., Wang Y., Wang C., Luo X. (2018). High-Resolution Grayscale Image Hidden in a Laser Beam. Light Sci. Appl..

[28] Zang X.F., Zhu Y.M., Mao C.X., Guo X.G., Gong H.H., Liang J.Q., Luo X. (2019). Manipulating Terahertz Plasmonic Vortex Based on Geometric and Dynamic Phase. Adv. Opt. Mater..

[29] Li G., Kang M., Chen S., Zhang S., Pun E.Y., Cheah K.W., Li J. (2013). Spin-Enabled Plasmonic Metasurfaces for Manipulating Orbital Angular Momentum of Light. Nano Lett..

[30] Ling X., Zhou X., Yi X., Luo H., Wen S., Liu Z., Chen J., Hao Y., Qiu C. (2015). Giant Photonic Spin Hall Effect in Momentum Space in a Structured Metamaterial with Spatially Varying Birefringence. Light Sci. Appl..

[31] Luo W., Xiao S., He Q., Sun F., Zhou L. (2015). Photonic Spin Hall Effect with Nearly 100% Efficiency. Adv. Opt. Mater..

[32] Shaltout A., Liu J., Kildishev A., Shalaev V. (2015). Photonic Spin Hall Meta-Lens for On-Chip Chiroptical Spectroscopy. Optica.

[33] Zhou J., Qian H., Hu G., Wang Y., Yang Y., Zhao T. (2018). Broadband Photonic Spin Hall Meta-Lens. ACS Nano.

[34] Zang X.F., Mao C.X., Guo X.G., Gong H.H., Liang J.Q., Luo X. (2018). Polarization-Controlled Terahertz Super-Focusing. Appl. Phys. Lett..

[35] Chen X., Huang L., Mühlenbernd H., Li G., Bai B., Tan Q., Jin G., Zentgraf T., Zhang S. (2012). Dual-Polarity Plasmonic Metalens for Visible Light. Nat. Commun..

[36] Chen X., Chen M., Mehmood M.Q., Huang L., Wen D., Li G., Zheng G., Zhang S., Chen X. (2015). Longitudinal Multifoci Metalens for Circularly Polarized Light. Adv. Opt. Mater..

[37] Shrestha S., Overvig A.C., Lu M., Stein A., Yu N., Kymissis I., Loncar M., Capasso F. (2018). Broadband Achromatic Dielectric Metalenses. Light Sci. Appl..

